# Cohort Profile Update: 2015 Pelotas (Brazil) Birth Cohort Study－follow-ups from 2 to 6–7 years, with COVID-19 impact assessment

**DOI:** 10.1093/ije/dyae048

**Published:** 2024-04-12

**Authors:** Joseph Murray, Otavio Amaral de Andrade Leão, Thaynã Ramos Flores, Flavio Fernando Demarco, Luciana Tovo-Rodrigues, Isabel O Oliveira, Adriane Arteche, Cauane Blumenberg, Andréa Dâmaso Bertoldi, Marlos Rodrigues Domingues, Mariangela Freitas Silveira, Pedro Curi Hallal

**Affiliations:** Postgraduate Program in Epidemiology, Federal University of Pelotas, Pelotas, Brazil; Human Development and Violence Research Centre (DOVE), Federal University of Pelotas, Pelotas, Brazil; Postgraduate Program in Epidemiology, Federal University of Pelotas, Pelotas, Brazil; Department of Kinesiology and Community Health, University of Illinois Urbana-Champaign, Urbana-Champaign, IL, USA; Postgraduate Program in Epidemiology, Federal University of Pelotas, Pelotas, Brazil; Postgraduate Program in Epidemiology, Federal University of Pelotas, Pelotas, Brazil; Postgraduate Program in Epidemiology, Federal University of Pelotas, Pelotas, Brazil; Human Development and Violence Research Centre (DOVE), Federal University of Pelotas, Pelotas, Brazil; Postgraduate Program in Epidemiology, Federal University of Pelotas, Pelotas, Brazil; Postgraduate Program in Psychology, Pontifical Catholic University of Rio Grande do Sul, Porto Alegre, Rio Grande do Sul, Brazil; Postgraduate Program in Epidemiology, Federal University of Pelotas, Pelotas, Brazil; Causale Consultoria, Pelotas, Rio Grande do Sul, Brazil; Postgraduate Program in Epidemiology, Federal University of Pelotas, Pelotas, Brazil; College of Physical Education, Federal University of Pelotas, Pelotas, Rio Grande do Sul, Brazil; Postgraduate Program in Epidemiology, Federal University of Pelotas, Pelotas, Brazil; Department of Kinesiology and Community Health, University of Illinois Urbana-Champaign, Urbana-Champaign, IL, USA

**Keywords:** Birth cohort, Brazil, child development, physical activity, violence

Key Features

- The 2015 Pelotas Birth Cohort is a population-based study in Pelotas city, Brazil. It originally aimed to investigate life-course determinants of health and development, and investigate time trends comparing with earlier birth cohorts in the same city. The 4275 participants were assessed at birth and previously followed at ages 3 and 12 months.
- Here we present details of new follow-ups at ages 2, 4 and 6–7 years, a COVID-19 impact study at age 5 years, and a nested randomized trial of two parenting programmes.
- New areas of research include violence and psychosocial development, stress, sleep patterns and impacts of the COVID-19 pandemic. New biomarkers have been collected, including hair cortisol concentration and genetic data, and new detailed assessments of the caregiving environment and child development have been made in recent follow-ups. The nested PIÁ trial evaluated effects of two group-based parent-training programmes on parenting and child development.
- Follow-up rates were 95.4% (*n* = 4014) at age 2 years, 95.4% (*n* = 4010) at 4 years and 92.0% (*n* = 3867) at 6–7 years. The web-based COVID-19 impact study included 2183 participants (56.6%). The PIÁ trial of parenting programmes includes 369 mother-child dyads.
- For collaboration proposals refer to our website [https://www.epidemio-ufpel.org.br] or contact the corresponding author [j.murray@doveresearch.org].

## The original cohort

The 2015 Pelotas (Brazil) Birth Cohort is a prospective study of all children born between 1 January and 31 December 2015 to women living in Pelotas city.^1^ Pelotas is a relatively poor city in Southern Brazil; see Table 1 for comparisons between Pelotas and Brazil on several socioeconomic indicators, infant mortality rates and violence. The 2015 cohort is the fourth in a series of similar cohort studies in Pelotas, which included children born in 1982, 1993 and 2004. The 2015 cohort was the first to include an assessment during pregnancy. The original aims were to investigate early life exposures for health outcomes, with special attention to physical activity and social inequalities. The original cohort profile^1^ described follow-ups in pregnancy, at birth and at ages 3 and 12 months. Two nested randomized trials of an exercise intervention in pregnancy^2^ and an infant-sleep intervention^3^ were also described.

**Table 1.** Comparison of selected sociodemographic, health and violence indicators between Pelotas city and Brazil

**Indicator** ^a^	Pelotas	Brazil
Human Development Index	0.739	0.727
Education: % of adults aged ≥18 years who completed primary school	58.0	54.9
Income: gross domestic product per capita in 1 year (R$)	21 553	29 466
Inequality: Gini index	0.54	0.60
Infant mortality: rate per 1000 live births	13.3	12.4
Homicides: rate per 100 000	33.5	28.4

a Data for income, infant mortality, and homicide are for 2015; data for Human Development Index and education are for 2010. Human Development Index, Education, Income, and Inequality data are from Instituto Brasileiro de Geografia e Estatística [http://www.atlasbrasil.org.br/consulta/planilha] and [https://www.ibge.gov.br/estatisticas/economicas/contas-nacionais]. Infant mortality and homicide data are from Sistema de Informação sobre Mortalidade, source DATASUS [https://datasus.saude.gov.br/mortalidade-desde-1996-pela-cid-10].

## What is the reason for the new data collection?

There were four main reasons to conduct new follow-ups at 2 years, 4 years, 5 years and 6–7 years: (i) to monitor life-course determinants of health as children moved into middle childhood; (ii) to examine time trends, comparing with previous Pelotas cohort studies; (iii) to establish new research on psychosocial development and violence, given the very high rates of violence in Brazil; (iv) to understand the impacts of the COVID-19 pandemic on cohort families and children, particularly given the very prolonged closure of schools in Brazil. Additionally, a nested randomized trial aimed to evaluate two parenting programmes that the local government planned to make public policy towards improving nurturing care in the population.

## What will be the new areas of research?

Beyond the original broad study aims and its focus on physical activity, several new areas of research are being investigated. Violence is a major health and social problem in Brazil, representing the leading cause of death among children and adolescents.^4^ A new core focus of the cohort is to understand the effects of violence on development, and determinants of behaviour problems implicated in later violence. This involves the study of complex environmental and psychological processes, as well as biological mechanisms. The new PIÁ trial, nested in the cohort, also examines effects of two parenting programmes as preventive interventions that are modest in cost and thus potentially scalable—both became public policy after the trial.

Other new areas of research aim to understand the effects of sleep patterns on child development and health, genetic influences and oral health. Stress is also being investigated, with repeated collection of hair samples to measure cortisol. We additionally aim to monitor the impact of the COVID-19 pandemic on children’s health and development. Pelotas schools were closed for over 1 year, and we have demonstrated that adverse consequences of the pandemic were particularly acute for poorer families.^5^ History of COVID-19 infection and vaccination were measured using antibody tests and questionnaires soon after the pandemic.

## Who is in the cohort?

**Figure 1.**
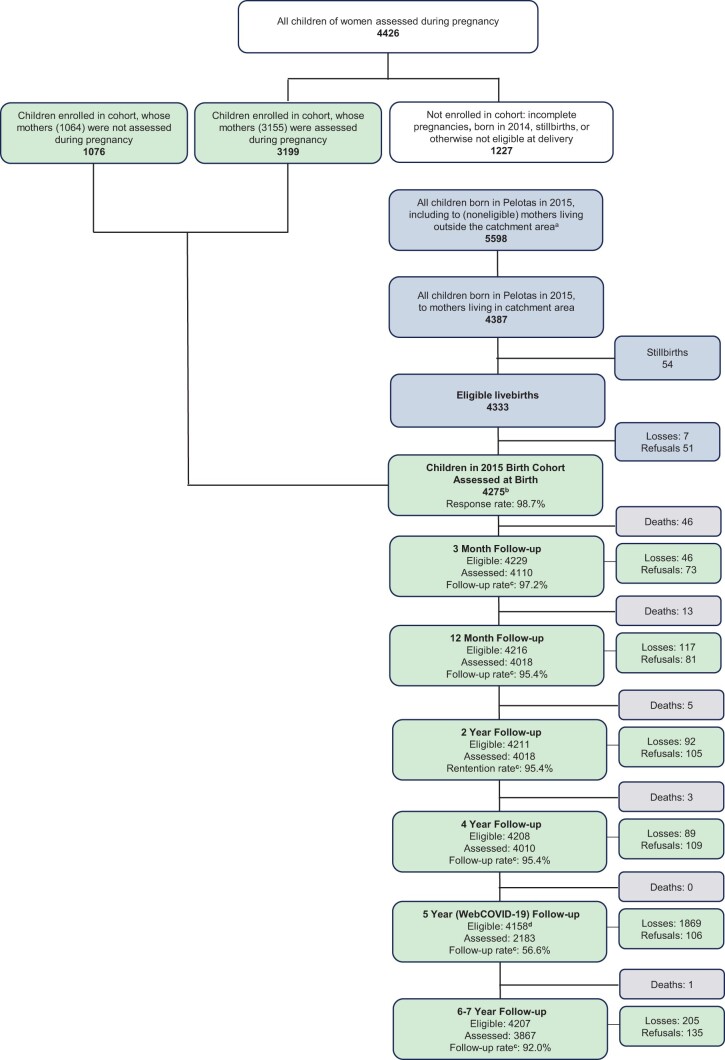
Flowchart of recruitment and participation in the 2015 Pelotas Birth Cohort Study. ^a^Cohort study catchment area, Pelotas urban area, Jardim America and Colônia Z3 (see Hallal *et al*. 2018^1^). ^b^Total of 4164 singletons, 108 twins, 3 triplets born to 4219 mothers. ^c^All live children were considered eligible for reassessment at each follow-up. As in all previous Pelotas birth cohort studies, follow-up rates are calculated as (4275 less number losses less number refusals)/4275. ^d^Only firstborn children among multiple births were eligible for this assessment. WebCOVID-19 is the name of the follow-up assessment completed by internet during the COVID-19 pandemic, when cohort children were aged 5 years. Some descriptions of the prenatal assessments and children identified as born in 2015 in this flowchart have been clarified, compared with a previous presentation (Hallal *et al*., 2018).^1^ Note also that in some publications we have referred to 4329 births occurring in 2015, which refers to all children included in the cohort (4275) as well as the 54 stillbirths who were not so included

New follow-ups were completed in 2017 (age 2), 2019 (age 4), 2020 (age 5, COVID-19 impact assessment, and 2021–22 (ages 6–7), when all participants were sought. Figure 1 shows an updated flowchart of initial recruitment and participation in all follow-ups. Table 2 shows characteristics of mothers and children at birth, for the whole cohort (*N*  =  4275) and for participants assessed in each of the follow-ups from ages 2 to 6–7 years. Generally, retention was high (92.0% to age 6–7) and participants assessed in recent waves are very similar to the whole cohort. However, there was significant non-participation in the web-based COVID-19 assessment in 2020 (56.6% follow-up rate), with participating mothers being more highly educated (72.8% had ≥9 years of schooling vs 65.2% in the whole cohort) and having slightly higher family income.

**Table 2.** Characteristics of mothers and children originally enrolled in the 2015 Pelotas Birth Cohort and participants included in recent follow-ups

Characteristic at birth	Original cohort	2 years	4 years	**5 years (WebCOVID-19** ^a^ **)**	6–7 years
No. of participants	4275	4014	4010	2183	3867
Maternal age (years)					
<19	431 (10.1%)	400 (10.0%)	406 (10.1%)	187 (8.6%)	397 (10.3%)
19–34	3210 (75.1%)	3025 (75.4%)	3015 (75.2%)	1675 (76.7%)	2909 (75.2%)
≥35	633 (14.8%)	589 (14.7%)	588 (14.7%)	320 (14.7%)	560 (14.5%)
Maternal education (years of completed schooling)					
0	17 (0.4%)	16 (0.4%)	17 (0.4%)	5 (0.2%)	16 (0.4%)
1–4	374 (8.8%)	340 (8.5%)	336 (8.4%)	105 (4.8%)	328 (8.5%)
5–8	1095 (25.6%)	1036 (25.8%)	1044 (26.0%)	484 (22.2%)	1007 (26.1%)
≥9	2788 (65.2%)	2621 (65.3%)	2612 (65.2%)	1589 (72.8%)	2514 (65.0%)
Wealth index (quintile)					
Poorest	824 (20.0%)	771 (19.9%)	773 (20.0%)	333 (15.6%)	748 (20.0%)
Second	829 (20.1%)	787 (20.3%)	794 (20.5%)	427 (20.0%)	774 (20.7%)
Third	820 (19.9%)	776 (20.0%)	775 (20.0%)	440 (20.6%)	755 (20.2%)
Fourth	823 (19.9%)	766 (19.8%)	772 (19.9%)	467 (21.8%)	739 (19.8%)
Wealthiest	831 (20.1%)	776 (20.0%)	759 (19.6%)	471 (22.0%)	719 (19.3%)
Family income (minimum wages)^b^					
<1.1	498 (12.4%)	463 (12.2%)	461 (12.2%)	201 (9.6%)	448 (12.3%)
1.1 to <3.1	1891 (47.1%)	1787 (47.3%)	1799 (47.7%)	973 (46.7%)	1754 (48.3%)
3.1 to <6.1	1064 (26.5%)	1006 (26.6%)	1003 (26.6%)	591 (28.4%)	947 (26.0%)
6.1 to <10.1	307 (7.6%)	283 (7.5%)	281 (7.5%)	170 (8.1%)	268 (7.4%)
≥10.1	256 (6.4%)	240 (6.4%)	228 (6.0%)	150 (7.2%)	218 (6.0%)
Parity					
1	2136 (50.0%)	2012 (50.1%)	2002 (49.9%)	1160 (53.2%)	1950 (50.5%)
2	1320 (30.9%)	1238 (30.9%)	1241 (31.0%)	679 (31.1%)	1187 (30.7%)
≥3	817 (19.1%)	762 (19.0%)	765 (19.1%)	343 (15.7%)	728 (18.8%)
Type of delivery					
Normal	1489 (34.8%)	1410 (35.1%)	1413 (35.2%)	744 (34.1%)	1360 (35.2%)
Caesarean section	2786 (65.2%)	2604 (64.9%)	2597 (64.8%)	1439 (65.9%)	2507 (64.8%)
Child sex					
Male	2164 (50.6%)	2030 (50.6%)	2028 (50.6%)	1119 (51.3%)	1953 (50.5%)
Female	2111 (49.4%)	1984 (49.4%)	1982 (49.4%)	1064 (48.7%)	1914 (49.5%)
Child birthweight (g)					
<2500	428 (10.1%)	383 (9.6%)	384 (9.6%)	182 (8.3%)	372 (9.6%)
2500–3499	2717 (63.8%)	2573 (64.1%)	2570 (64.1%)	1413 (64.8%)	2490 (64.4%)
≥3500	1113 (26.1%)	1055 (26.3%)	1053 (26.3%)	587 (26.9%)	1003 (26.0%)

a WebCOVID-19 is the name of the follow-up assessment completed by internet during the COVID-19 pandemic, when cohort children were aged 5 years. There were also assessments completed during pregnancy and at 3 and 12 months postpartum, but those have been described in a previous publication.^1^

b In some previous publications [e.g.^1^], the definition of intervals between income groups was mis-specified, and those have been corrected here.

### Follow-up at 2 years

Up to the 2-year follow-up, 64 deaths were identified. In 2017, all surviving cohort members (*N*  =  4211) were sought for assessment at age 2 years, by telephoning primary caregivers, contacts on social media and visiting last known addresses. The study was also advertised in local newspapers and radio, and on Facebook; 4014 children were assessed (follow-up rate 95.4%) at average age 24.0 months (SD  =  7.3).

Unlike previous postnatal assessments which were home based, at 2 years the families were invited to the university research clinic, according to date of birth, where most participants were assessed. Assessments were also made at home (14.1%) and by phone/internet (2.3%) to increase participation among families who lived in other cities/states, and for those with a strong preference for home assessment.

### Follow-up at 4 years

We aimed to assess all live cohort members in 2019 at age 4 years using similar procedures to the 2-year follow-up, as well as using WhatsApp, Facebook and Instagram searches and consulting the Unified Health System (*CadSUS WEB)* to locate participants. Three further deaths were identified up to age 4 years. Of 4208 surviving participants, 4010 were assessed (follow-up rate 95.4%) at average age 45.5 months (SD  =  2.6). Among non-responders at age 4 (*n*  =  198), about half (*n*  =  105) had also previously declined to participate at age 2. Assessments were conducted at the research clinic, apart from 8.5% assessments conducted at home and 2.5% by phone/internet.

### Follow-up at 5 years (WebCOVID-19)

During the COVID-19 pandemic, public sector schools and kindergartens were closed in Pelotas city from 17 March 2020 until April 2021, when in-person learning was slowly phased back. Between May and September 2020, we sought to assess all live singletons and firstborn twins in the cohort (*N * = * *4158) via an internet-based questionnaire, to identify pandemic-related family experiences and child adjustment, which was called the WebCOVID-19 assessment. Mothers or caregivers were invited to participate via e-mail, social media and telephone. For those without internet access, a telephone interview was offered, and 3% of respondents completed the questionnaire by phone. In all, 2163 assessments were made (56.6% response rate), and Table 1 shows that respondents were more highly educated and had a slightly higher family income than the whole cohort. Children included in this follow-up had an average age of 60.5 months (SD  =  3.6).

### Follow-up at 6–7-years

We aimed to assess all cohort members in 2021–22 at age 6–7 years. Similar procedures to the 4-year follow-up were used. Furthermore, study details were disseminated in some private schools to locate participants. Only one more death was identified until the 6–7-year follow-up. Out of the 4207 remaining participants, 3867 were assessed in 2021–22 (92.0%), at an average age of 81.6 months (SD  =  3.8). Assessments were conducted in the research clinic, apart from for 18.8% which were conducted at home and 5.2% by phone/internet.

### PIÁ trial follow-ups

The PIÁ trial aims to evaluate two group-based parenting programmes implemented in 2018 (child age 3 years), among 369 mother-child cohort pairs. The programmes were ACT: Raising Safe Kids, and a dialogue book-sharing programme. Details of interventions, ethical approval, power calculations and pre-registry are in the trial protocol.^6^ Trial outcomes are parenting practices and child development, behaviour and stress. A baseline assessment was conducted before randomization to one of the two parenting interventions or control group. At 1-month post-intervention, 369 (100%) of the participants were reassessed. During the age 4 cohort follow-up, 368 (99%) of the trial sample were reassessed, and at ages 6–7 years, 366 (99%) were reassessed.

## What has been measured?

Table 3 shows the general socioeconomic and health assessments made at ages 2, 4, 5 and 6–7 years, and Table 4 shows mental health and psychosocial assessments made in these follow-ups, which form a major new line of research in the cohort (Supplementary Tables S1 and S2, available as Supplementary data at *IJE* online provide details on measures). As part of the psychosocial assessments, parent-child interactions during three tasks were filmed, for the whole cohort at age 4 years, and subsequently coded by (blinded) psychologists regarding parenting sensitivity, parent-child reciprocity, emotional tone, coercive behaviours and other parenting dimensions. These films have also been transcribed for content analyses. Hair cortisol concentration and COVID-19 test data are available and genetic material is stored for analyses. In the PIÁ trial sub-sample (*n*  =  369), further measures of parenting and child development were used, as detailed in another publication.^6^

**Table 3.** Socioeconomic and health measures used in recent follow-ups of the 2015 Pelotas Birth Cohort

**Assessment** ^a^	2 years	4 years	5 years (WebCOVID-19)	6–7 years
General health and social questionnaires				
Sociodemographic characteristics	✔	✔	✔	✔
Employment	✔	✔		✔
Breastfeeding	✔	✔		
Diet	✔	✔		✔
Medicine use	✔	✔		✔
Vaccination	✔	✔		✔
Health care use	✔	✔		✔
Health care expenditures	✔			✔
Physical activity questionnaire	✔	✔	✔	✔
Child care arrangements	✔	✔		✔
Child screen time	✔	✔	✔	✔
Child sleep characteristics	✔	✔		✔
Child oral health	✔	✔		
Maternal characteristics	✔	✔	✔	✔
Maternal health and contraceptive use	✔	✔		✔
Child physical examinations and biological samples				
Saliva for genetic analyses	✔			✔
Hair cortisol		✔		✔
Resting heart rate		✔		✔
Heart rate before and after stress				✔
Head circumference	✔	✔		
Anthropometry	✔	✔		✔
Body composition (DXA, BodPod)				✔
Physical activity (accelerometry)	✔	✔		✔
Oral health examination		✔		
COVID-19 antibody test				✔
Maternal physical examinationss and biological samples				
Hair cortisol		✔		
Anthropometry	✔	✔		✔
Physical activity (accelerometry)	✔	✔		✔
COVID-19 pandemic specific questions				
Financial difficulties, welfare support			✔	
Food insecurity			✔	
Child fears about pandemic			✔	
School activities			✔	
Social distancing, isolation			✔	
Maternal social distancing, isolation			✔	

BodPod, air displacement plethysmography system; DXA, dual X-ray absorptiometry.

a Measures used are shown in Supplementary Table S1, available as Supplementary data at *IJE* online.

**Table 4.** Mental health and psychosocial assessments in recent follow-ups of the 2015 Pelotas Birth Cohort

**Assessment** ^a^	2 years	4 years	5 years (WebCOVID-19)	6–7 years
Observed (filmed) parent-child interactions				
Responsive interactions		✔		
Book-sharing interactions		✔		
‘Don’t touch’ interactions		✔		
Child-based assessments				
Overall development	✔	✔		
Intelligence				✔
Executive functions		✔		✔
Theory of mind		✔		✔
Prosocial behaviour		✔		✔
Emotion recognition		✔		
Hostile attribution bias		✔		
Moral judgements				✔
Perceived social support				✔
Mother-reported measures				
Parenting behaviours	✔	✔	✔	✔
Child stimulation activities	✔	✔		✔
Child mental health		✔	✔	✔
Child aggression	✔	✔	✔	✔
Child callous: unemotional traits		✔		
Child stressful life events		✔		✔
Child victimization		✔		✔
Maternal risk taking		✔		
Maternal substance use		✔		
Maternal anxiety	✔		✔	✔
Maternal depression	✔	✔	✔	✔
Maternal PTSD				✔
Maternal self-control		✔		
Maternal hostile attribution bias		✔		
Maternal perceptions of social-legal fairness and social standing		✔		
Maternal perceptions of trust				✔
Maternal perceptions of police violence				✔
Maternal perceived norms about violence				✔
Maternal social support				✔
Maternal adverse childhood experiences		✔		
Maternal experiences of intimate partner violence		✔		✔
Maternal stress		✔		
Parental relationship conflict	✔	✔	✔	✔
Parental antisocial behaviour		✔		
Parental crime				✔
Neighbourhood violence and social cohesion		✔		

PTSD, post-traumatic stress disorder.

a Instruments used are shown in Supplementary Table S2, available as Supplementary data at *IJE* online.

## What has it found? Key findings and publications

Nearly 100 articles have been published so far on a range of health and child development topics. A supplement in the *International Journal of Epidemiology* (Vol. 48, Supplement 1) described time trends across the four Pelotas cohorts (1982, 1993, 2004 and 2015). During the 33-year period between the oldest and youngest cohorts, there were positive changes in social and environment determinants of health, including income, education, fertility and home environment. Socioeconomic inequality reduced. There were also major improvements in maternal and child health, such as rates of breastfeeding and reduced stunting. However, other indicators worsened, including maternal hypertension, diabetes, overweight and obesity, and prevalence of caesarean sections and preterm births, admissions to neonatal intensive care units and infant overweight. Full immunization coverage in the first year of life decreased from 80.9% in 1982 to 77.2% in 2015.^7^

### Maternal health

The prevalence of unplanned pregnancies decreased between the 1993 and 2015 cohorts but remained high (52.2% in 2015)^8^; the prevalence of unmet need for modern contraceptives was 10.7% in the 2015 cohort.^9^ Both inadequate and excessive gestational weight gain in pregnancy were common: 30.6% and 35.9%, respectively.^10^ Lower socioeconomic status was strongly associated with maternal depression in the first 2 years postpartum (slope index of inequality, SII, at 3 months: −17.5).^11^ Overall, 23% of mothers experienced persistently high depressive symptoms until the age 2 follow-up,^12^ which was associated with greater risk for child hospitalization (prevalence ratio, PR  =  1.96 for the high chronic depression trajectory vs low depression trajectory).^13^

### Physical activity

Physical activity (PA) research is a key focus of the cohort since its inception. Key findings include: (i) maternal PA in the third trimester was protective against preterm birth (PR  =  0.58)^14^; (ii) meeting PA recommendations during pregnancy was associated with less movement limitation due to low back pain (odds ratio, OR  =  0.60)^15^; (iii) child PA predicted positive neurodevelopment at age 4 years (β  =  2.22 for children with high PA trajectories vs low).^16^

The PAMELA trial^2^ was described in the original cohort profile^1^ and aimed to evaluate effects of an exercise intervention during pregnancy. No benefits were found regarding preeclampsia, preterm birth and postpartum depression,^17^^,^^18^ possibly because of low intervention adherence (<40%). The intervention was associated with higher child language (standardized mean difference, SMD  =  0.23) at age 2 years and cognitive scores at age 4 (SMD  =  0.22).^19^ Despite this specific benefit, the main trial finding was that exercise during pregnancy did not have negative associations with health outcomes.

### Nutrition and oral health

Children’s consumption of ultraprocessed food increased between ages 2 and 4 years,^20^ a period when the prevalence of child overweight also increased from 7.6% to 12.9%.^20^ At age 4 years, 5.4% of children were classified as obese.^21^ Dental caries at age 4 were associated with both prolonged breastfeeding (≥24 months breastfeeding: PR  =  1.82) and high consumption of ultraprocessed foods (PR  =  1.16),^22^ as well as a pattern of increasing sugar consumption between 3 months and 4 years (PR  =  1.48 compared with low sugar consumption).^23^

### Child development

Poorer child development (<10th percentile on overall development scores at age 2) was associated with maternal pre-pregnancy underweight for girls (OR  =  2.14); for boys, poorer language (OR  =  1.59) and cognition (OR  =  1.59) scores were predicted by excessive maternal gestational weight gain.^24^ Cognitive development at age 2 years was positively associated with child care attendance from ages 1 to 2 years (β  =  2.44 compared with children never in child care).^25^ Child use of screens increased from an average of 3.4 h per day to 4.4 h at age 4 years, between the 2004 and 2015 cohorts, but no important association was observed between screen time and child development outcomes.^26^ A quasi-experimental evaluation of a large-scale home-visiting programme (Primeira Infância Melhor) showed that, although the programme had no overall effects on child development at age 4 years, benefits were observed among families who had enrolled during pregnancy.^27^

### Violence

Almost a fifth of cohort mothers reported experiencing physical/verbal abuse or disrespect at hospital during childbirth, and this was associated with postnatal depression, particularly for physical abuse (OR  =  2.28).^28^ Maternal adverse childhood experiences (ACEs) were associated with increased exposure to intimate partner violence (PR  =  4.9 comparing women with 5+ ACEs vs no ACEs) when cohort children were aged 4 years, as well as cohort children’s exposure to maltreatment (PR  =  3.8).^29^ The co-occurrence of intimate partner violence and child maltreatment was strongly associated with neighbourhood violence, absence of the child’s biological father, parental antisocial behaviours and mental health problems.^30^ Intimate partner violence had important associations with parenting practices up to 3 years later; for example, parental exposure to emotional violence at child age 2 predicted more coercive behaviours when children were aged 6–7 (SMD  =  0.22).^31^

### COVID-19 and mental health

There were marked inequalities regarding the impact of the COVID-19 pandemic on cohort families. Thus, poorer families were at far greater risk of experiencing serious financial problems, food shortages, increased conflict in adult relationships, parenting problems and child worries about food availability. For example, among those in the lowest quintile of family income before the pandemic, 43.1% experienced food insecurity compared with 6.2% of the highest quintile families. In turn, these difficulties were associated with increases in mental health problems for both caregivers and children; e.g. food insecurity associated with maternal depression (β  =  0.19) and anxiety (β  =  0.18) and child emotional problems (β  =  0.12) during the pandemic, adjusting for pre-pandemic symptoms and confounders (βs are standardized regression coefficients for mental health outcome scores in linear regression models).^5^

### Other findings

The Sleep Trial^3^ was described in the original cohort profile.^1^ It assessed the efficacy of an educational intervention on child night-time sleep duration, and no effects were found.^32^ Stress measured in terms of cortisol levels from hair samples were related to sociodemographic factors^33^ but not children’s physical activity.^34^

## What are the main strengths and weaknesses?

The main strengths include the high initial response rate at birth (98.7%) and follow-up rate to age 6–7 years (92.0%). The study includes high-quality measures across many domains of health and psychosocial development. Data can be compared with similar earlier birth cohorts, enabling examination of time trends as well as life-course processes.

It is a limitation that, although information about children’s fathers is available from maternal reports, there are limited data collected directly from fathers. The response rate in the WebCOVID-19 study in 2020 was low (56.6%), and these data require weighting to make better population estimates.^5^ Data linked to home addresses are unavailable, but they are currently being collected to enable geocoded spatial analyses. DNA from saliva samples is currently stored, but genotyping has not been conducted. Funding is being sought for these analyses and the next follow-up, planned when children are 11 years old.

## Can I get hold of the data? Where can I find out more?

We have successfully collaborated with investigators from many countries worldwide, including partnerships with researchers in the UK, Canada, Uganda, USA, Australia, Norway, Uruguay, Chile and Portugal. We also collaborate with Brazilian institutions and participate in the Brazilian RPS (Ribeirão Preto-Pelotas-São Luiz) birth cohorts consortium.^35^ Exchange of doctoral or postdoctoral fellows between other institutions and Pelotas is very welcome: see [http://www.epidemio-ufpel.org.br/site/content/home-en/] or e-mail the investigators involved in the research areas of interest. The questionnaires and interviewer guides from all follow-up visits are available at [http://www.epidemio-ufpel.org.br/site/content/coorte_2015-en/index.php]. We welcome collaborative research proposals to use data from any of the follow-ups, particularly involving local researchers and postgraduate students. Data access is given via submission of a paper proposal to a publications committee, and contact about potential collaboration and access to data can be addressed to [j.murray@doveresearch.org].

## Ethics approval

The 2015 cohort assessments were approved by the research ethics committees of the Federal University of Pelotas (School of Physical Education 0-4 years, #26746414.5.0000.5313; Faculty of Medicine: Genetic data #62251516.6.0000.5317; PIÁ Trial ##2.602.769; 4 years psychological assessments #03837318.6.0000.5317; COVID-19 pandemic follow-ups #31179020.7.0000.5313; 6–7 years follow-up #51789921.1.0000.5317) and participants provided written informed consent.

## Supplementary Material

dyae048_Supplementary_Data

## Data Availability

See ‘Can I get hold of the data?’ above.
