# Supplementary material for: Cohort Profile Update: 2015 Pelotas (Brazil) Birth Cohort Study－follow-ups from 2 to 6–7 years, with COVID-19 impact assessment
Source: Int J Epidemiol. 2024 Apr 12;53(3):dyae048. doi: 10.1093/ije/dyae048 (PMC11014790; doi:10.1093/ije/dyae048)
Supplement: dyae048_Supplementary_Data [file dyae048_supplementary_data.pdf]

Cohort Profile Update: 2015 Pelotas (Brazil) Birth Cohort Study — Follow-ups from 2 to 6-7 years, with Covid-19 impact assessment

Supplementary material

Table S1. Socioeconomic and health instruments used in recent follow-ups of the 2015 Pelotas Birth Cohort

	**Instrument (citation)**	**2 years**	**4 years**	**5 years (WebCOVID-19)**	**6-7 years**
**General health and social questionnaires**					
Sociodemographic characteristics	Pelotas study questions	✓	✓	✓	✓
Employment	Pelotas study questions	✓	✓		✓
Breastfeeding	Pelotas study questions	✓	✓		
Diet	Pelotas study questions – based on food consumption marker form of Food and Nutrition Surveillance System [1]	✓	✓		✓
Medicine use	Pelotas study questions	✓	✓		✓
Vaccination	Pelotas study questions	✓	✓		✓
Health care use	Pelotas study questions	✓	✓		✓
Health care expenditures	Pelotas study questions	✓			✓
Physical activity questionnaire	Pelotas study questions	✓	✓	✓	✓
Childcare arrangements	Pelotas study questions	✓	✓		✓
Child screen time	Pelotas study questions	✓	✓	✓	✓
Child sleep characteristics	Pelotas study questions	✓	✓		✓
Child oral health	Pelotas study questions	✓	✓		
Maternal characteristics	Pelotas study questions	✓	✓	✓	✓
Maternal health and contraceptive use	Pelotas study questions	✓	✓		✓
**Child physical exams & biological samples**					
Saliva for genetic analyses	OG-500 - DNA Genotek®	✓			✓
Hair cortisol	ELISA technique using the Salivary Cortisol High Sensitivity Immunoassay Kit (Cat# 1-3002, Salimetrics, Pennsylvania), adapted as in Martins et al. [2]. The ELISA plate reader SpectraMax®190 (Molecular Devices, U.S) was used for cortisol detection, and final cortisol concentrations are expressed in pg/mg.		✓		✓
Resting heart rate	OMRON® HEM- 705CPINT		✓		✓
Heart rate before and after stress	Xiaomi Mi Smart Band 6®				✓

	Instrument (citation)	2 years	4 years	5 years (WebCOVID-19)	6-7 years
Head circumference	CESCORF® flexible steel measuring tape, 2m long and 6mm wide	✓	✓		
Anthropometry	TANITA® 17 scale, model UM-080, with a maximum capacity of 150 kg and an accuracy of 100g, used to measure child's weight.<br>A fixed stadiometer from the Harpenden® brand, with a maximum height of 2.06m and an accuracy of 1mm to measure child's standing height and sitting height - torso measurement.	✓	✓		✓
Body composition	Bod Pod® and DXA: enCORE-based X-ray Bone Densitometer - Lunar Prodigi model – GE Healthcare® brand.				✓
Physical activity (Accelerometry)	*ActiGraph®,* model *wGT3X-BT*. Devices were attached to child's left wrist, with a 24h use protocol. The number of days of use varied according to device availability and child age.	✓	✓		✓
Oral health exam	Clinical examination of: visible plaque, dental trauma, occlusion, erosion, developmental defects in enamel (DDE), dental caries and problems related to odontogenic infection (PUFA). Hygiene condition was determined using the IHO-S, modified for the primary dentition [3].		✓		
COVID-19 antibody test	ELISA test – blood collection using filter paper				✓
**Maternal physical exams and biological samples**					
Maternal hair cortisol	ELISA technique using the Salivary Cortisol High Sensitivity Immunoassay Kit (Cat# 1-3002, Salimetrics, Pennsylvania), adapted as in Martins et al. [2]. The ELISA plate reader SpectraMax®190 (Molecular Devices, U.S) was used for cortisol detection, and final cortisol concentrations are expressed in pg/mg.		✓		
Anthropometry	TANITA® 17 scale, model UM-080, with a maximum capacity of 150 kg and an accuracy of 100g, used to measure mother's weight.	✓	✓		✓

	Instrument (citation)	2 years	4 years	5 years (WebCOVID-19)	6-7 years
	A fixed stadiometer from the Harpenden® brand, with a maximum height of 2.06m and an accuracy of 1mm to measure mother's standing height and sitting height - torso measurement.				
Physical activity (Accelerometry)	*ActiGraph®,* model *wGT3X-BT*	✓	✓		✓
**COVID-19 pandemic specific questions**					
Financial difficulties, welfare support	Pelotas study questions			✓	
Food insecurity	Pelotas study questions			✓	
Child fears about pandemic	Pelotas study questions			✓	
School activities	Pelotas study questions			✓	
Social distancing-isolation	Pelotas study questions			✓	
Maternal social distancing-isolation	Pelotas study questions			✓	

Notes. WebCOVID-19 is the name of the follow-up assessment completed by internet during the COVID-19 pandemic, when cohort children were aged 5 years. DXA: Dual-Energy X-ray Absorptiometry; PUFA index: This index records the consequences of an untreated carious lesion (P–Pulpal involvement, U-Ulceration, F-Fistula and A-abscess); IHOS: Simplified Oral Hygiene Index; pg/mg: picogram/milligram; ELISA: Enzyme-Linked Immunosorbent Assay.

Table S2. Mental health and psychosocial instruments in recent follow-ups of the 2015 Pelotas Birth Cohort

	Instrument [citation]	2 years	4 years	5 years WebCOVID-19	6-7 years
**Filmed parent-child interactions**					
Responsive Interactions	Filmed Responsive Interactions Task [4]		✓		
Book-sharing Interactions	Filmed Book-sharing Task [5]		✓		
"Don't Touch" Interactions	Filmed Don't Touch Task [6]		✓		
**Child-based assessments**					
Overall child development	The Intergrowth-21st Neurodevelopment Assessment (INTER-NDA) *2y* [7], Battelle's Development Inventory – screening version *4y* [8]	✓	✓		
Intelligence	Wechsler Intelligence Scale for Children - 4th edition (WISC-IV) [9]				✓
Executive functions	Early Years Toolbox (EYT) Card Sorting [10], EYT Go/No-Go *4y* [10], Marshmallow test (Gratification Delay task) *4y* [11], Modified emotional Stroop colour-naming task *6-7y* [12]		✓		✓
Theory of mind	Sally-Anne False-belief Task [13]		✓		✓
Prosocial behaviour	Filmed Help Task *4y* [14], Dictator Game [15]		✓		✓
Emotion recognition	Affect Knowledge Task – "Puppets" [16]		✓		
Social Information Processing - Hostile attribution bias	The Social Information Processing Interview – Preschool Version (SIPI-I) [17]		✓		
Moral judgements	Moral judgement tasks - distributive justice / reasoning about vignettes [18, 19]				✓
Perceived social support	Pelotas study questions				✓
**Mother-reported measures**					
Parenting behaviours	Parent and Family Adjustment Scales (PAFAS) [20]; Parent-Child Conflict Tactics Scale (CTSPC) *6-7y* [21]	✓	✓	✓	✓
Child stimulation activities	Pelotas study questions [22]	✓	✓		✓

	**Instrument [citation]**	**2 years**	**4 years**	**5 years WebCOVID-19**	**6-7 years**
Child mental health	Strengths and Difficulties Questionnaire (SDQ) [23], Development and Well-Being Assessment (DAWBA) *6-7y* [24]		✓	✓	✓
Child aggression	*Etude longitudinale du development des enfants du Quebec* (ELDEQ) questionnaire [25]	✓	✓	✓	✓
Child callous-unemotional traits:	Inventory of Callous-Unemotional Traits short-form (SF-ICU) [26]		✓		
Child stressful life events	Pelotas study questions		✓		✓
Child victimisation	Juvenile Victimization Questionnaire, 2nd edition, Screener Sum Version (JVQ-R2) [27]		✓		✓
Maternal risk taking	Balloon Analogue Risk Task (BART) [28]		✓		
Maternal substance use	Alcohol, Smoking and Substance Involvement Screening Test (ASSIST) [29]		✓		
Maternal anxiety	Generalized Anxiety Disorder (GAD-7) [30]	✓		✓	✓
Maternal depression	Edinburgh Postnatal Depression Scale (EPDS) [31]	✓	✓	✓	✓
Maternal PTSD	Posttraumatic Stress Disorder Checklist for DSM-5 (PCL-5) [32]				✓
Maternal self-control	Brief Self Control Scale (BSCS) [33]		✓		
Maternal hostile attribution bias	Parental Hostile Attribution Questionnaire (Parental-HAQ) [34]		✓		
Maternal perceptions of social-legal fairness and social standing	Questions from prior studies [35-39]		✓		
Maternal perceptions of trust	Adapted OECD measure [40]				✓
Maternal perceptions of police violence	Pelotas study questions				✓
Maternal perceived norms about violence	Pelotas study questions				✓
Maternal social support	Pelotas study questions				✓
Maternal adverse childhood experiences	Adverse Childhood Experiences International Questionnaire (ACE-IQ) [41]		✓		
Maternal experiences of intimate partner violence	WHO Questionnaire [42]		✓		✓

	**Instrument [citation]**	**2 years**	**4 years**	**5 years WebCOVID-19**	**6-7 years**
Maternal stress	Stress-Producing Life Events Inventory (SPLEI) [43], Perceived Stress Scale reduced (PSS10) [44]		✓		
Parental relationship conflict	Questions from prior study [45]	✓	✓	✓	✓
Parental antisocial behaviour	Mini International Neuropsychiatric Interview - version 5.0 (MINI) [46]		✓		
Parental crime	Pelotas study questions				✓
Neighbourhood violence & social cohesion	Questions from prior studies [47, 48]		✓		

Notes. WebCOVID-19 is the name of the follow-up assessment completed by internet during the COVID-19 pandemic, when cohort children were aged 5 years. PTSD: Post-traumatic stress disorder.

Supplement References

1. Ministério da Saúde. Orientações para Avaliação de Marcadores de Consumo Alimentar na Atenção Básica. Brasilia, DF: Ministério da Saúde; 2023.
2. Martins RC, Tovo-Rodrigues L, Oliveira I, Blumenberg C, Bertoldi AD, Silveira MF, et al. Determinants of hair cortisol in preschool children and their mothers: A Brazilian birth cohort study. Psychoneuroendocrinology. 2023;**150**:106027.
3. Greene JC, Vermillion JR. The Simplified Oral Hygiene Index. J Am Dent Assoc. 1964;**68**:7-13.
4. Schneider A, Rodrigues M, Falenchuk O, Munhoz TN, Barros AJD, Murray J, et al. Cross-Cultural Adaptation and Validation of the Brazilian Portuguese Version of an Observational Measure for Parent–Child Responsive Caregiving. International Journal of Environmental Research and Public Health. 2021;**18**(3):1246.
5. Murray L, De Pascalis L, Tomlinson M, Vally Z, Dadomo H, MacLachlan B, et al. Randomized controlled trial of a book-sharing intervention in a deprived South African community: effects on carer–infant interactions, and their relation to infant cognitive and socioemotional outcome. J Child Psychol Psychiatry. 2016;**57**(12):1370-9.
6. Kochanska G, Aksan N. Mother-Child Mutually Positive Affect, the Quality of Child Compliance to Requests and Prohibitions, and Maternal Control as Correlates of Early Internalization. Child Dev. 1995;**66**(1):236-54.
7. Murray E, Fernandes M, Newton CRJ, Abubakar A, Kennedy SH, Villar J, et al. Evaluation of the INTERGROWTH-21st Neurodevelopment Assessment (INTER-NDA) in 2 year-old children. PLoS One. 2018;**13**(2):e0193406.
8. Newborg J, Stock JR, Wnek L, Guidubaldi J, Svinicki J. Battelle Developmental Inventory. Rolling Meadows, IL: Riverside; 2005.
9. Wechsler D, Golombok S, Rust J. Weschler Intelligence Scale for Children (Third Edition). The Psychological Corporation: London; 1992.
10. Howard SJ, Melhuish E. An Early Years Toolbox for Assessing Early Executive Function, Language, Self-Regulation, and Social Development: Validity, Reliability, and Preliminary Norms. Journal of psychoeducational assessment. 2017;**35**(3):255-75.
11. Mischel W, Ebbesen EB. Attention in delay of gratification. J Pers Soc Psychol. 1970;**16**(2):329-37.
12. Processing bias and anxiety in primary school children: A modified emotional stroop colour-naming task using pictorial facial expressions [press release]. Germany: Pabst Science Publishers2004.
13. Baron-Cohen S, Leslie AM, Frith U. Does the autistic child have a “theory of mind” ? Cognition. 1985;**21**(1):37-46.
14. Dunfield KA, Kuhlmeier VA. Classifying prosocial behavior: children's responses to instrumental need, emotional distress, and material desire. Child Dev. 2013;**84**(5):1766-76.
15. Benenson JF, Pascoe J, Radmore N. Children's altruistic behavior in the dictator game. Evolution and Human Behavior. 2007;**28**(3):168-75.
16. Denham SA, Couchoud EA. Young preschoolers' understanding of emotions. Child Study Journal, . 1990;**20**(3):171-92.
17. Ziv Y, Sorongon A. Social information processing in preschool children: Relations to sociodemographic risk and problem behavior. J Exp Child Psychol. 2011;**109**(4):412-29.
18. Krcmar M, Valkenburg PM. A Scale to Assess Children's Moral Interpretations of Justified and Unjustified Violence and Its Relationship to Television Viewing. Communication Research. 1999;**26**(5):608-34.
19. Huppert E, Cowell JM, Cheng Y, Contreras-Ibáñez C, Gomez-Sicard N, Gonzalez-Gadea ML, et al. The development of children's preferences for equality and equity across 13 individualistic and collectivist cultures. Dev Sci. 2019;**22**(2):e12729.
20. Sanders MR, Morawska A, Haslam DM, Filus A, Fletcher R. Parenting and Family Adjustment Scales (PAFAS): Validation of a Brief Parent-Report Measure for Use in Assessment of Parenting Skills and Family Relationships. Child Psychiatry Hum Dev. 2014;**45**(3):255-72.

21. Reichenheim ME, Moraes CL. [Portuguese-language cross-cultural adaptation of the Parent-Child Conflict Tactics Scales (CTSPC), an instrument used to identify parental violence against children]. Cad Saude Publica. 2003;**19**(6):1701-12.
22. Barros AJ, Matijasevich A, Santos IS, Halpern R. Child development in a birth cohort: effect of child stimulation is stronger in less educated mothers. Int J Epidemiol. 2010;**39**(1):285-94.
23. Goodman R. The Strengths and Difficulties Questionnaire: A research note. J Child Psychol Psychiatry. 1997;**38**(5):581-6.
24. Goodman R, Ford T, Richards H, Gatward R, Meltzer H. The Development and Well-Being Assessment: Description and initial validation of an integrated assessment of child and adolescent psychopathology. J Child Psychol Psychiatry. 2000;**41**(5):645-55.
25. Girard L-C, Tremblay RE, Nagin D, Côté SM. Development of Aggression Subtypes from Childhood to Adolescence: a Group-Based Multi-Trajectory Modelling Perspective. J Abnorm Child Psychol. 2019;**47**(5):825-38.
26. Hawes SW, Byrd AL, Henderson CE, Gazda RL, Burke JD, Loeber R, et al. Refining the parent-reported inventory of callous-unemotional traits in boys with conduct problems. Psychol Assess. 2014;**26**(1):256-66.
27. Finkelhor D, Hamby SL, Turner H, Ormrod R. The Juvenile Victimization Questionnaire: 2nd Revision (JVQ-R2). Durham, NH: Crimes Against Children Research Center; 2011.
28. Lejuez CW, Read JP, Kahler CW, Richards JB, Ramsey SE, Stuart GL, et al. Evaluation of a behavioral measure of risk taking: the Balloon Analogue Risk Task (BART). J Exp Psychol Appl. 2002;**8**(2):75-84.
29. WHO ASSIST Working Group. The Alcohol, Smoking and Substance Involvement Screening Test (ASSIST): development, reliability and feasibility. Addiction. 2002;**97**(9):1183-94.
30. Spitzer RL, Kroenke K, Williams JBW, Löwe B. A Brief Measure for Assessing Generalized Anxiety Disorder: The GAD-7. Arch Intern Med. 2006;**166**(10):1092-7.
31. Cox JL, Holden JM, Sagovsky R. Detection of Postnatal Depression: Development of the 10-item Edinburgh Postnatal Depression Scale. The British Journal of Psychiatry. 1987;**150**(6):782-6.
32. OsÓRio FL, Silva TDAD, Santos RGDOS, Chagas MHN, Chagas NMS, Sanches RF, et al. Posttraumatic Stress Disorder Checklist for DSM-5 (PCL-5): transcultural adaptation of the Brazilian version. Archives of Clinical Psychiatry (São Paulo). 2017;**44**.
33. Tangney JP, Baumeister RF, Boone AL. High Self-Control Predicts Good Adjustment, Less Pathology, Better Grades, and Interpersonal Success. J Pers. 2004;**72**(2):271-324.
34. Halligan SL, Cooper PJ, Healy SJ, Murray L. The attribution of hostile intent in mothers, fathers and their children. J Abnorm Child Psychol. 2007;**35**(4):594-604.
35. Tyler TR, Fagan JA, editors. Legitimacy and Cooperation: Why Do People Help the Police Fight Crime in Their Communities?2010.
36. Arsenio WF, Willems C. Adolescents' conceptions of national wealth distribution: Connections with perceived societal fairness and academic plans. Dev Psychol. 2017;**53**(3):463-74.
37. Kay AC, Jost JT. Complementary justice: effects of "poor but happy" and "poor but honest" stereotype exemplars on system justification and implicit activation of the justice motive. J Pers Soc Psychol. 2003;**85**(5):823-37.
38. Fagan J, Piquero AR. Rational Choice and Developmental Influences on Recidivism Among Adolescent Felony Offenders. Journal of empirical legal studies. 2007;**4**(4):715.
39. Adler NE, Epel ES, Castellazzo G, Ickovics JR. Relationship of subjective and objective social status with psychological and physiological functioning: preliminary data in healthy white women. Health psychology : official journal of the Division of Health Psychology, American Psychological Association. 2000;**19 6**:586-92.
40. Brezzi M, González S, Nguyen D, Prats M. An updated OECD framework on drivers of trust in public institutions to meet current and future challenges. 2021.
41. Organization WH. Adverse Childhood Experiences International Questionnaire (ACE-IQ) 2020.

42. World Health Organization. WHO multi-country study on women's health and domestic violence against women : initial results on prevalence, health outcomes and women's responses. Geneva: World Health Organization; 2005.
43. Rizzini M, Santos AMd, Silva AAMd. Evidence of validity of the Stress-Producing Life Events (SPLE) instrument. Revista de Saúde Pública. 2018;**52**.
44. Cohen S, Kamarck T, Mermelstein R. A global measure of perceived stress. J Health Soc Behav. 1983;**24**(4):385-96.
45. Hooley JM, Teasdale JD. Predictors of relapse in unipolar depressives: expressed emotion, marital distress, and perceived criticism. J Abnorm Psychol. 1989;**98**(3):229-35.
46. Sheehan DV, Lecrubier Y, Sheehan KH, Amorim P, Janavs J, Weiller E, et al. The Mini-International Neuropsychiatric Interview (M.I.N.I.): the development and validation of a structured diagnostic psychiatric interview for DSM-IV and ICD-10. J Clin Psychiatry. 1998;**59 Suppl 20**:22-33.
47. Sampson RJ, Graif C. Neighborhood Social Capital as Differential Social Organization: Resident and Leadership Dimensions. Am Behav Sci. 2009;**52**(11):1579-605.
48. Mujahid MS, Diez Roux AV, Morenoff JD, Raghunathan T. Assessing the measurement properties of neighborhood scales: from psychometrics to ecometrics. Am J Epidemiol. 2007;**165**(8):858-67.
